# Preparation, Magnetic and Mechanical Properties of Fe/Ni-Based Amorphous Fibers

**DOI:** 10.3390/ma17153733

**Published:** 2024-07-28

**Authors:** Shuang Su, Wenjie Zhao, Yagnesh Shadangi, Jiapeng Zhang, Zhiliang Ning, Jianfei Sun, Yongjiang Huang

**Affiliations:** 1 School of Materials Science and Engineering, Harbin Institute of Technology, Harbin 150001, China; sushuang@hit.edu.cn (S.S.); nuczhaowenjie@163.com (W.Z.); b18031665138@163.com (J.Z.); jfsun_hit@263.net (J.S.); 2 Department of Materials Science & Metallurgical Engineering, Indian Institute of Technology Bhilai, Durg 491001, Chhattisgarh, India; yshadangi@iitbhilai.ac.in; 3 CITIC Dicastal Co., Ltd., Qinhuangdao 066011, China

**Keywords:** amorphous fibers, magnetic property, statistical analysis, tensile tests

## Abstract

In this study, we successfully fabricated Fe_61_Zr_10_Co_5_Mo_7_W_2_B_15_ and Ni_61_Nb_19_._2_Ta_19_._8_ amorphous fibers (AFs) using the melt-extraction method. This method ensured a rapid cooling, uniform quality, minimal defects, and superior performance. Magnetic property analysis revealed that the Fe-based AFs exhibited a single-slope magnetization curve characteristic of paramagnetic or diamagnetic materials, while the Ni-based AFs displayed a rectangular curve with low magnetic hysteresis, typical of ferromagnetic materials. The axial saturation magnetization of as-prepared Ni-based AFs is ~1.5 × 10^−7^ emu/g, with a coercivity of about 85 Oe. The statistical analysis of tensile tests indicated that Ni-based AFs possess a higher fracture threshold of 2440 ± 199 MPa and a reliability of 14.7, demonstrating greater material safety and suitability for high-performance applications. As opposed to Ni-based AFs, Fe-based AFs present a fracture threshold and of 1582 ± 692 MPa and a reliability 4.2. Moreover, under cyclic loading conditions, Ni-based AFs exhibited less residual deformation and superior elastic recovery with a fracture strength of 2800 MPa. These findings highlight the potential of Ni-based AFs for advanced engineering applications, particularly where high strength, durability, and excellent magnetic properties are required, paving the way for their integration into next-generation technologies.

## 1. Introduction

Amorphous alloys, characterized by their inability to nucleate and crystallize due to extremely rapid cooling rates from their molten state, inherently lack grain boundaries and dislocations in their structure [1]. This results in atoms being arranged in a disordered manner across three dimensions, indicative of their long-range disorder yet short-range order [2]. Amorphous fibers (AFs), notably those with micron-scale diameters produced through specialized processes, demonstrate exceptional properties including superior strength, hardness, toughness, and notable electromagnetic and wear resistance [3]. The emergence of these materials in the 1980s marked the beginning of extensive research and applications, highlighting their significant role in enhancing the functionality of devices across a wide range of fields such as security, metallurgy, healthcare, micro-devices, electronics, and information technologies [4,5,6]. Specific studies, such as those by Wang et al. [7] on the tensile properties of cold-drawn Co-based AFs, which showed a fracture strength of 4000 MPa and a plasticity of 1.6%, and by Yi et al. [8] on the preparation of Pd_40_Cu_30_Ni_10_P_20_ amorphous nanowires, indicated an inverse correlation between tensile plasticity and fiber diameter.

Advances in processing technologies have resulted in substantial progress in the fabrication of bulk amorphous materials and one-dimensional AFs [9,10,11]. Among many prevalent wire-drawing techniques, the melt-extraction method can readily achieve high-performance AFs [12]. However, it involves high-speed rotating wheels dipping into the melt and encompasses a complex process of momentum, energy, and mass transfer [13]. Factors such as wheel rotation speed, vertical feed rate, melt superheat, and alloy composition play critical roles in the metallurgical quality of fibers [14]. Furthermore, great variations in the micro-scale dimensions of the fibers, along with the associated size effects, critically affect the microstructural attributes and properties of the AFs [15].

In this study, AFs with compositions such as Fe_61_Zr_10_Co_5_Mo_7_W_2_B_15_ (at. %) and Ni_61_Nb_19_._2_Ta_19_._8_ (at. %) were selected due to their higher crystallization temperatures, which makes them promising for high-temperature applications [16,17]. The two compositions can form compounds with magnesium alloy and are likely to achieve superior interface bonding and improved microscopic properties [18]. There has been limited research on the preparation techniques for these types of AFs. Fundamental research on Fe/Ni-based AFs is of great importance for the composite development of magnesium alloys and AFs, as well as for the broader application of AFs. The Fe-based and Ni-based AFs were fabricated by the melt extraction technique. The mechanical and magnetic properties of melt-extracted AFs were studied, in order to explore their wider applications.

## 2. Experimental Methods

### 2.1. Samples Preparation

The melt-extraction technique [12] was utilized to fabricate one-dimensional fibers from rod samples with compositions of Fe_61_Zr_10_Co_5_Mo_7_W_2_B_15_ (at. %) and Ni_61_Nb_19_._2_Ta_19_._8_ (at. %). A master alloy rod was positioned within a boron nitride (BN) crucible, which had its inner hole finely polished to ensure a precise fit between the crucible and the alloy rod. The crucible was placed beneath the Mo wheel, with the gap between them adjusted for optimal performance. To achieve an environment conducive to the fabrication of high-quality fibers, the furnace was initially vacuumed to 10^−3^ Pa. Subsequently, an argon atmosphere was introduced to provide gas shielding. Upon completion of these steps, the induction melting source of the system was engaged alongside the wheel, which had been set to a predefined rotation speed. The molten alloy was then engaged by the wedge-shaped rim of the Mo wheel, achieving a cooling rate as high as 10^6^ K/s. The fibers obtained have a diameter of ~50 μm.

### 2.2. Structural Characterizations

Surface morphologies of melt-extracted AFs were examined using a scanning electron microscope (SEM, Helios Nanolab 600i, FEI, Hillsboro, OR, USA). The elemental compositions of the AFs were detected using energy dispersive spectroscopy (EDS). The instruments were operated at an acceleration voltage of 20 kV, emission current of 90 μA, EDS working distance of 10 mm, and a scanning probe current of 50 μA. Transmission electron microscopy (TEM) characterization of the AFs was performed using JEM-2100 Talos equipment (FEI, New York, NY, USA).

### 2.3. Magnetic Performance Tests

The magnetic properties of AFs were tested using a physical property measurement system (PPMS, Dynacool-14T, Quantum Design, New York, NY, USA) which consists of a mainframe along with various measurement and extension options. For this study, the vibrating sample magnetometer (VSM) option from the series of products was chosen to perform magnetization tests on the AFs. The tests were conducted at 300 K, and the direction of the tests was aligned with the axial direction of the AFs.

### 2.4. Mechanical Property Tests

Tensile test specimens of fiber materials were prepared according to the ASTM D3379-75 standard [19]. The mechanical properties of AFs were tested using an Instron 5943 micro tensile tester. The applied strain rate is 4.2 × 10^−4^ s^−1^. To ensure accurate statistical analysis, thirteen Fe-based specimens and eleven Ni-based specimens with the same diameter were used for the tensile tests. For the tensile-tensile cyclic test, the samples were subjected to tensile loading in an algorithm test consisting of maximum loads of 0.5 N, 1.0 N, 1.5 N, 2.0 N, and 2.5 N. An important aspect in axial cyclic testing is the uniformity of stress and strains in the specimen gage section. This type of test was performed using the same equipment presented above.

## 3. Results and Discussions

### 3.1. Structural Characterizations

**Figure 1:**
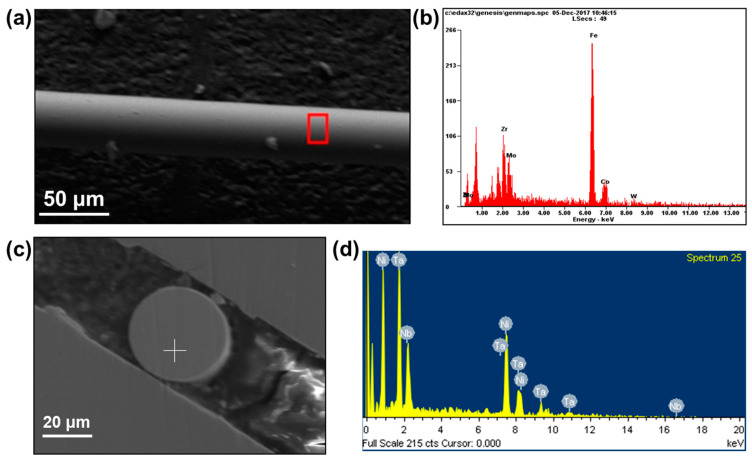
(**a**) SEM image of Fe_61_Zr_10_Co_5_Mo_7_W_2_B_15_ AF. (**b**) EDS result of red box in (**a**). (**c**) SEM image of Ni_61_Nb_19_._2_Ta_19_._8_ AF. (**d**) EDS result of the marked position in (**c**).

EDS analysis was performed on Fe-based AFs using SEM. The results are shown in Figure 1. The areas selected for EDS analysis are indicated by the boxes in Figure 1a, and the results are presented in Figure 1b and Table 1. As seen, there is no clear macroscopic segregation, indicating a reasonable preparation process. EDS analysis was also conducted on the cross-section of Ni-based Afs. Spot scanning locations are shown in Figure 1c. The results of the EDS analysis are presented in Figure 1d and Table 2. It can be observed that the composition of the selected sample areas closely matches the nominal chemical composition of Fe_61_Zr_10_Co_5_Mo_7_W_2_B_15_ and Ni_61_Nb_19_._2_Ta_19_._8_.

**Figure 2:**
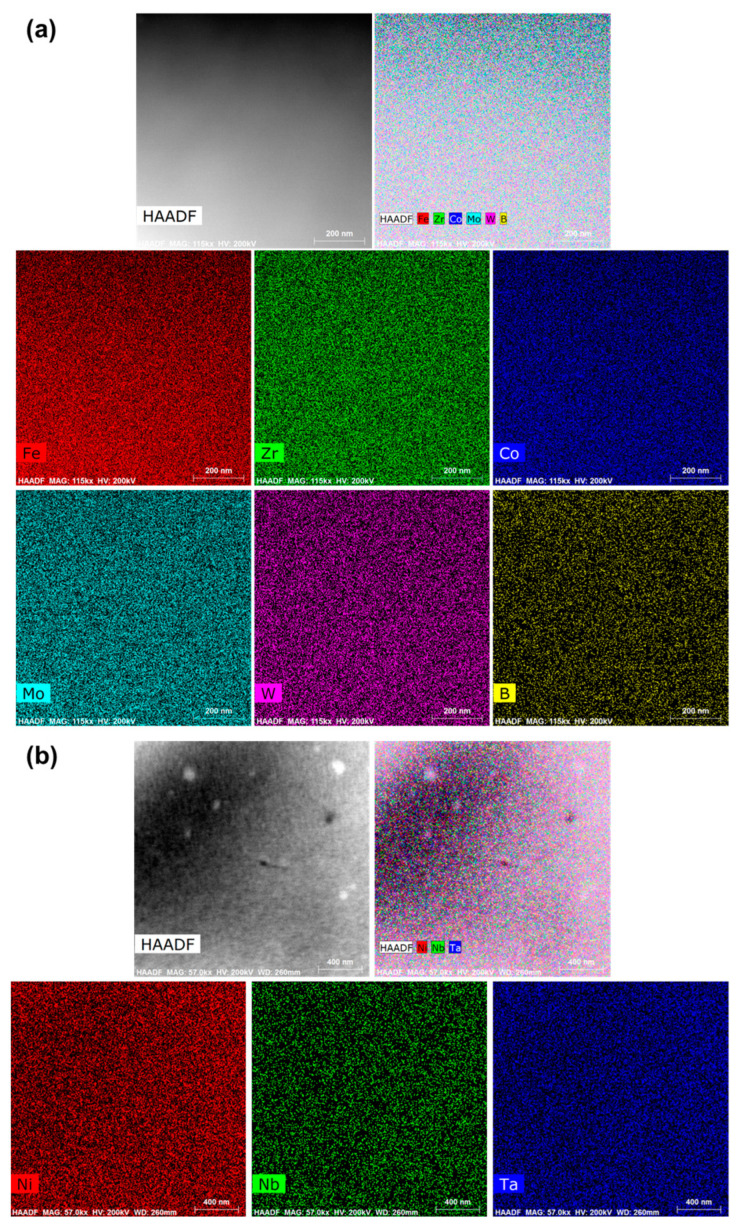
Scanning transmission electron microscopy (STEM)- High angle annular dark field (HAADF) image and the EDS elemental maps of (**a**) Fe_61_Zr_10_Co_5_Mo_7_W_2_B_15_ and (**b**) Ni_61_Nb_19_._2_Ta_19_._8_ AFs.

To observe the uniformity of the composition, area scanning based on TEM was performed on the samples. The results are shown in Figure 2. The AFs produced by the melt-extraction method exhibit a high degree of compositional uniformity, with no evident micro-segregation.

### 3.2. Magnetic Properties

**Figure 3:**
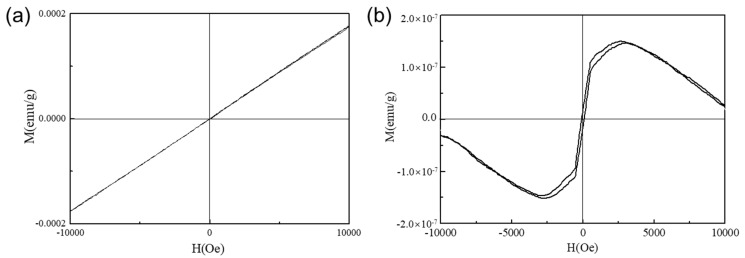
Magnetization curves of (**a**) Fe_61_Zr_10_Co_5_Mo_7_W_2_B_15_ and (**b**) Ni_61_Nb_19_._2_Ta_19_._8_ AFs.

Due to their excellent electromagnetic properties and thus potential engineering applications, Fe-based, and Ni-based AFs have received widespread attention [20,21]. In this study, VSM was used to measure the magnetic properties of the melt-extracted Fe- and Ni-based AFs due to their high sensitivity and precision. The corresponding magnetization curves are shown in Figure 3. It can be observed from Figure 3a that, when the magnetic field is applied along the axial direction, there is no obvious hysteresis phenomenon. The hysteresis loop can be regarded as a curve with a constant slope. Saturation was not reached during measurement—both the coercivity (*H*_c_) and remnant magnetization (*B*_r_) were approximately zero. The melt-extracted Fe-based AFs exhibit characteristics of paramagnetism or diamagnetism. However, it is not possible to determine the specific type of magnetism simply from the hysteresis loop. However, it can be preliminarily determined that they are not ferromagnetic materials. As can be observed in Figure 3b, the melt-extracted Ni-based fibers exhibit a clear hysteresis phenomenon, with the hysteresis loop having a shape like a flattened rectangle, indicating low magnetic loss and characteristics of ferromagnetic materials. The *M*_a_ of the melt-extracted Ni-based AFs is approximately 1.5 × 10^−7^ emu/g, and the *H*_c_ is about 85 Oe. As the magnetic intensity is applied beyond 5000 Oe, the magnetization decreases, suggesting that the melt-extracted Ni-Nb-Ta AFs exhibit weak magnetism.

The magnetization curves of Ni-based AFs are not completely symmetrical. That means the coercivity displayed on both sides of the hysteresis loop is not equal, resulting in a shift of the loop. However, there is no change of the hysteresis loop, implying that the slopes on either side of the loop are almost the same. A few researchers and co-workers have reported the asymmetricity in the hysteresis loop due to annealing, magnetic treatment and mechanical working [22,23,24]. Chen et al. [25] reported a shift in the hysteresis loop along with the magnetic field for Co-based amorphous alloys annealed above 180 °C. It is generally believed that such a shift in Co-based amorphous alloys after annealing can be attributed to the precipitation of a small amount of a second phase or segregation with higher coercivity, which then exerts a bias magnetization on the surrounding amorphous matrix. In another work, Liu et al. [26] discerned greater shift in the hysteresis loop resulting from magnetic field annealing. In previous research on FeCuNbSiB alloy [27], a significant shift in the hysteresis loop was also found after annealing in a DC magnetic field. This was attributed to the fluctuations in the alloy composition and inhomogeneities.

Unlike Fe-based AFs, Ni-based AFs exhibit a significant asymmetry in their magnetization process due to several key factors. Firstly, the internal stress induced by the rapid quenching process in Ni-based AFs leads to structural anisotropy, affecting the magnetization process differently in various directions and resulting in an asymmetric magnetization curve [28]. In contrast, Fe-based AFs generally exhibit better structural stability, leading to more symmetrical magnetization curves [29]. Additionally, Ni-based AFs are more prone to forming short-range order or clusters, which introduce magnetocrystalline anisotropy and further contribute to the asymmetry in the magnetization curve [30]. Surface characteristics also play a crucial role in the magnetic properties of Ni-based AFs. Surface irregularities, oxidation, and other surface-related phenomena can cause variations in the magnetization process, leading to curve asymmetry [31]. Fe-based AFs, on the other hand, typically have more stable surface conditions, resulting in more symmetrical magnetization behavior [32]. Additionally, the magnetoelastic coupling effect in Ni-based AFs can induce mechanical strains when there are changes in magnetization. These strains, in turn, affect the magnetization process, causing an asymmetric response. Fe-based AFs tend to exhibit weaker magnetoelastic coupling effects, contributing to their relatively symmetrical magnetization curves [33]. In a word, the combination of internal stress, surface characteristics, and magnetoelastic coupling in Ni-based AFs results in an asymmetric magnetization curve, in stark contrast to the more symmetrical behavior observed in Fe-based AFs.

The magnetic properties of amorphous materials have been extensively studied, with a focus on their applications in electronic and information technologies. Zhukova et al. [34] investigated the stress-induced anisotropy in Co-rich glass-coated microwires, demonstrating how internal stresses influence magnetic behavior. Similarly, our findings indicate that Ni-based AFs exhibit low magnetic hysteresis and ferromagnetic properties, making them suitable for applications requiring precise magnetic control.

Ni-based AFs exhibit low magnetic hysteresis, high saturation magnetization, and excellent soft magnetic properties. These characteristics make them highly suitable for applications in high-performance electronic and information technology devices, such as magnetic sensors, transformers, and inductors. Their low coercivity and high permeability ensure efficient energy conversion and signal processing, which are critical for the development of miniaturized and high-frequency components in telecommunications and computing industries. Fe-based AFs, on the other hand, also possess valuable magnetic properties, including high saturation magnetization and excellent soft magnetic behaviors, although they generally exhibit higher coercivity and magnetic hysteresis compared to Ni-based AFs. These properties make Fe-based AFs suitable for applications where high magnetic field strengths are required, such as in magnetic cores for power transformers, inductors, and electric motors. The cost-effectiveness of Fe-based AFs further enhances their appeal for large-scale industrial applications.

### 3.3. Mechanical Properties

Gaining better understanding of the deformation behaviors of AFs is beneficial for widening their industrial applications. Generally, AFs exhibit a dispersed mechanical performance, which has a negative impact on their reliability. In this study, a series of tensile tests were performed on melt-extracted AFs. The corresponding stress-strain curves are shown in Figure 3a,b. The tensile behaviors of Fe-based and Ni-based specimens of uniform diameter were ascertained by tensile testing of ‘13’ and ‘11’ AFs, respectively, under the same strain rate. The standard deviations of the Fe- and Ni-based AFs are ±692 MPa and ±199 MPa, indicating more stable mechanical properties of Ni-based AFs.

Next, Weibull statistical analysis was carried out on the melt-extracted AFs in order to quantitatively assess the failure probability [35].

For the rank evaluation algorithm, we used a median rank formula based on the specific experimental data of AF samples for calculating the failure probability *P_i_*, namely

(1) $$P_{i}=\frac{i-0.3}{N+0.5}$$

where *i* is the sample number and *N* is the overall number of the testing samples, of which Fe-based AFs is 13, and Ni-based AFs is 11. The three-parameter model, due to its complexity in parameters, has various estimation and application methods, each resulting in different errors. The first step in these methods is essentially to linearize the process. That is, for *N* independently and identically distributed experimental data points, i.e., the fracture strengths (*σ*_(1)_, *σ*_(2)_, …, *σ*_(*N*)_), are arranged in ascending order to obtain a set of order statistics *σ*_1_ < *σ*_2_ < … < *σ_N_*. The probability of material failure under the applied stress *σ_i_* is given by the median rank formula. At this point, the Weibull function can be expressed as:

(2) $$P^{WB}=1-\exp[-\int_{V}{\left(\frac{\sigma -\delta }{\beta }\right)}^{\alpha }d_{V}]$$

Taking the double logarithm of the above equation yields the commonly used linearized formula:

(3) $$\ln\{\ln[\frac{1}{1-P^{WB}}]\}=\alpha \ln(\sigma -\delta )-\alpha \ln\beta$$

Generally, if the fracture strength *σ_i_* and the probability *P_i_* are directly fitted, a larger scale parameter *β* will be obtained. This might not be because of the specificity of the tensile fracture strength of amorphous samples only, but also possibly because the data dispersion is too great, leading to a larger scale parameter. The value of the scale parameter *β* depends not only on the size of the location parameter *α*, but also on the difference (*δ*-*μ*). For the sake of convenience in research, the natural logarithm of fracture stress is commonly used as the abscissa. Weibull parameter estimation is performed on *N* groups of experimental data (ln(*σ_i_*), ln[−ln(1 − *P_i_*)]), eliminating obvious outliers, resulting in the arrangement of fracture strength from smallest to largest as shown in the figure below.

**Figure 4:**
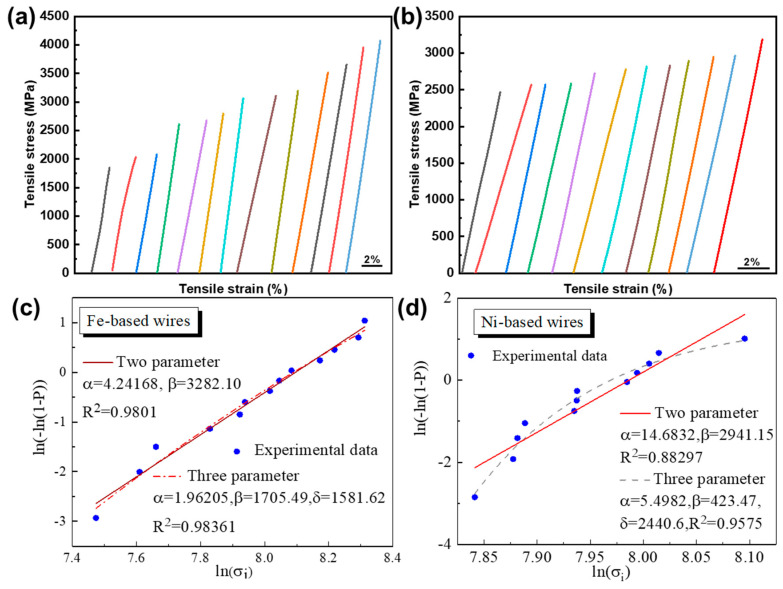
Tensile stress-strain curves of (**a**) Fe_61_Zr_10_Co_5_Mo_7_W_2_B_15_ and (**b**) Ni_61_Nb_19_._2_Ta_19_._8_ AFs and (**c**,**d**) their two- and three-parameter Weibull statistical analysis plots.

Fitting the data in Figure 4a,b with both three-parameter and two-parameter Weibull statistical analysis yields the results shown in Figure 4c,d. Figure 4c presents the parameter-fitting results for the fracture strength of Fe-based AFs, while Figure 4d shows the fitting results for the fracture strength of Ni-based AFs. The fitting curves are strictly convex and achieved good coefficient values, indicating that the fracture strength data of the as-prepared samples conform to Weibull statistical analysis.

For the two-parameter model, the moduli for Fe-based and Ni-based are 4.24 and 14.68, respectively, with fitting coefficients (*R*^2^) of 0.9801 and 0.88297, respectively. These fitting coefficients are lower than those for the corresponding three-parameter fits, indicating its lower fitting accuracy. The three-parameter model demonstrates a higher degree of fit. The stress threshold *δ* values are 1582 MPa and 2440 MPa, respectively, clearly indicating higher material safety for Ni-based materials. The parameter α, serving as an index to measure material reliability, suggests to some extent that the fracture reliability of melt-extracted AFs is higher than that of traditional brittle materials (*m* = 5), thereby offering higher safety. At the same time, there is a significant difference in safety between Ni-based and Fe-based materials. The higher modulus of Ni-based models indicates lower data dispersion, suggesting that Ni-based AFs have higher safe stress and a higher degree of safety, whereas Fe-based AFs, with a smaller *δ*, tend to undergo catastrophic fracture under low-stress conditions.

Mechanical properties, particularly fracture strength and reliability, are crucial for the industrial application of AFs. The work of Yi et al. [8] on Pd-based metallic glass nanowires highlights the inverse correlation between tensile plasticity and fiber diameter, a concept supported by our observations that Ni-based AFs have higher fracture thresholds and reliability compared to Fe-based AFs. This reliability is further quantified through Weibull statistical analysis, aligning with Elgueta and Kittl’s [36] approach to probabilistic control of material performance. Recent studies have emphasized the importance of compositional uniformity and structural integrity in amorphous materials. For instance, Abrosimova et al. [37] highlighted that the absence of micro-segregation in Al-based amorphous alloys significantly enhances their mechanical properties. Our study corroborates this by showing that the melt-extracted Fe- and Ni-based AFs exhibit uniform composition and minimal defects, contributing to their excellent performance.

**Figure 5:**
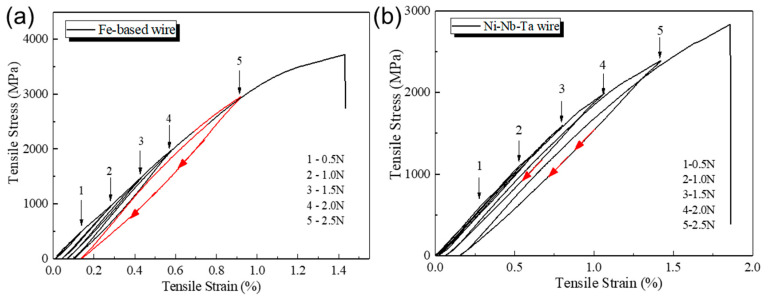
Cyclic loading stress–strain curves of (**a**) Fe_61_Zr_10_Co_5_Mo_7_W_2_B_15_ and (**b**) Ni_61_Nb_19_._2_Ta_19_._8_ AFs.

The cyclic loading curves of melt-extracted Fe- and Ni-based AFs are shown in Figure 5, with cyclic conditions maintained at 0.5 N, 1.0 N, 1.5 N, 2.0 N, and 2.5 N. These curves represent a single sample undergoing five cyclic loading tests. Compared to Ni-based fibers, Fe-based fibers in Figure 5a showed residual deformation from the second cycle of loading and maintained basically the same residual strain after each cycle. The strain hysteresis is not significant, with a good overlap of the loading-unloading curves in each cycle except for the fifth one. Even during the sixth (last) loading, when the stress reached the unloading point of the fifth cycle, the corresponding strain still matched well with the strain of the fifth cycle’s unloading point, with almost zero difference. When the stress reached 3300–3400 MPa, there was a noticeable change in the slope of the stress–strain curve. For Ni-based AFs, the red downward arrows in Figure 5b indicate the stress–strain paths during the fourth and fifth unloading. Under cyclic loading conditions, the fracture strength of the Ni-based AFs increased slightly, with a final fracture strength of about 2800 MPa, while Fe-based AF can reach up to 3712 MPa. During the first three cycles, the stress-strain curves almost overlapped, with a small amount of residual deformation and clear elastic deformation, allowing them to return to the initial position upon unloading. When the load reached 2.0 N and 2.5 N, during the fourth and fifth cyclic loadings, some residual deformation occurred, and the tensile curve showed clear deviation from linear deformation, with a brittle fracture still occurring at the break without a yield plateau. This indicates that the yield strength is located between the third and fourth loadings. Additionally, it should be noted that, while generating residual strain, the path of the stress-strain curve also deviates from the previous ones. When the strain is larger, the AFs endure higher stress, causing an increase in deviation. The cyclic loading tests in our study reveal significant differences between Ni-based and Fe-based AFs in terms of residual deformation and elastic recovery. This finding is consistent with the research by Wang et al. [7] who related residual stress and microstructure to the mechanical properties of cold-drawn Co-based amorphous microwires. Our results indicate that Ni-based AFs maintain superior elastic recovery under cyclic loading, making them more suitable for applications involving repetitive stress.

In conclusion, both Fe-based and Ni-based AFs offer valuable properties for various applications [38]. Ni-based AFs, with their superior magnetic and mechanical properties, are well-suited for high-performance and precision applications [39], while Fe-based AFs provide a cost-effective alternative for broader industrial use. The distinct advantages of each material highlight the value of the results obtained in this study, paving the way for future research and development in these promising areas.

## 4. Summary

In conclusion, Fe_61_Zr_10_Co_5_Mo_7_W_2_B_15_ and Ni_61_Nb_19_._2_Ta_19_._8_ AFs were successfully fabricated by a melt-extraction method. Their magnetic and mechanical properties were studied. The following conclusions can be drawn:

(1) The melt-extraction method involves a high cooling rate, producing AFs with uniform quality, fewer size defects, and excellent performance, and good forming capability for high-melting-point metals. The surface morphology of melt-extracted AFs is round and uniform in composition. AFs with a high glass-forming ability, such as Fe- and Ni-based compositions, are suitable for preparation by the melt-extraction method.

(2) There are differences in the magnetization curves of Fe-based/Ni-based AFs. Axial magnetization curve tests at room temperature for Fe- and Ni-based AFs indicate that the magnetization curve of Fe-based AFs presents a single slope, characteristic of paramagnetic or diamagnetic materials. Ni-based AFs exhibit a flattened rectangular shape in their magnetization curves, with low magnetic hysteresis loss, indicative of ferromagnetic material properties. The axial saturation magnetization of as-prepared Ni-based AFs is approximately 1.5 × 10^−7^ emu/g, with a coercivity of about 85 Oe.

(3) Statistical analysis of the fracture sample data of AFs has led to safety estimations for Fe- and Ni-based AFs. For the two-parameter Weibull statistical analysis, the moduli for Fe- and Ni-based AFs are 4.24 and 14.7, respectively, indicating a higher fracture reliability for Ni-based AFs. The three-parameter fitting results demonstrate a higher degree of fit, with fracture threshold values *δ* of 1582 ± 692 MPa (Fe-based) and 2440 ± 199 MPa (Ni-based), clearly indicating the higher material safety of Ni-based fibers.

## Figures and Tables

**Table 1:** EDS results of Fe_61_Zr_10_Co_5_Mo_7_W_2_B_15_ AFs.

Element	Weight %	Atomic %
Fe	58.08 ± 0.46	61.02 ± 0.05
Zr	15.02 ± 0.53	9.66 ± 0.26
Co	5.65 ± 0.47	5.63 ± 0.51
Mo	11.51 ± 0.18	7.04 ± 0.05
W	7.08 ± 0.88	2.26 ± 0.30
B	2.65 ± 0.11	14.39 ± 0.51

**Table 2:** EDS results of Ni_61_Nb_19_._2_Ta_19_._8_ AFs.

Element	Weight %	Atomic %
Ni	40.02 ± 0.21	60.96 ± 0.21
Nb	20.08 ± 0.10	19.32 ± 0.12
Ta	39.90 ± 0.14	19.72 ± 0.11

## Data Availability

Data are contained within the article.

## References

[1] Michalik S., Molcanova Z., Sulikova M., Kusnirova K., Jovari P., Darpentigny J., Saksl K. (2023). Structure and Physical Properties of Mg_93-x_Zn_x_Ca_7_ Metallic Glasses. Materials.

[2] Pierwola A., Lelito J., Krawiec H., Szucki M., Gondek L., Koziel T., Babilas R. (2024). Non-Isothermal Analysis of the Crystallization Kinetics of Amorphous Mg_72_Zn_27_Pt_1_ and Mg_72_Zn_27_Ag_1_ Alloys. Materials.

[3] Atzmon M., Ju J.D., Lei T. (2023). Structural Relaxation, Rejuvenation and Plasticity of Metallic Glasses: Microscopic Details from Anelastic Relaxation Spectra. Materials.

[4] Chen M., Zhu L., Chen Y., Dai S., Liu Q., Xue N., Li W., Wang J., Huang Y., Yang K. (2024). Effect of Chemical Composition on the Thermoplastic Formability and Nanoindentation of Ti-Based Bulk Metallic Glasses. Materials.

[5] Hong Y., Wang H., Li X., Zhong L., Chen H., Zhang Z., Cao P., Ritchie R.O., Wang J. (2023). Structural heterogeneity governing deformability of metallic glass. Matter.

[6] Ouyang D., Zhao L., Li N., Pan J., Liu L., Chan K.C. (2023). Atomistic investigation of modulating structural heterogeneities to achieve strength-ductility synergy in metallic glasses. Comput. Mater. Sci..

[7] Wang H., Qin F.X., Xing D.W., Cao F.Y., Wang X.D., Peng H.X., Sun J.F. (2012). Relating residual stress and microstructure to mechanical and giant magneto-impedance properties in cold-drawn Co-based amorphous microwires. Acta Mater..

[8] Yi J., Wang W.H., Lewandowski J.J. (2015). Sample size and preparation effects on the tensile ductility of Pd-based metallic glass nanowires. Acta Mater..

[9] Zhang Q., Li Q.K., Li M. (2015). Internal stress and its effect on mechanical strength of metallic glass nanowires. Acta Mater..

[10] Xie J., Pei C., Zhou J., Ding D., Feng T., Li H., Sun B. (2023). Tensile plasticity with enhanced *β*-relaxation in high-energy metallic glass wires. Sci. China Mater..

[11] Yi J. (2018). Fabrication and Properties of Micro- and Nanoscale Metallic Glassy Wires: A Review. Adv. Eng. Mater..

[12] Su S., Huang Y., Zhang J., Zhang L., Wang H., Ning Z., Sun J. (2022). Tensile Properties of Melt-Extracted and Annealed NiFe-Based Amorphous Metallic Fibers. Metals.

[13] Zhang X., Sun Y., Yan B., Zhuang X. (2023). Correlation of Magnetomechanical Coupling and Damping in Fe80Si9B11 Metallic Glass Ribbons. Materials.

[14] Zheng S.-H., Wang Q., Zhu L.-Z., Wang P.-J., Ding D., Tang B.-Z., Yu P., Yao J.-L., Xia L. (2023). Excellent Magnetocaloric Performance of the Fe87Ce13− xBx (x= 5, 6, 7) Metallic Glasses and Their Composite. Materials.

[15] Wang H., Xing D., Peng H., Qin F., Cao F., Wang G., Sun J. (2012). Nanocrystallization enabled tensile ductility of Co-based amorphous microwires. Scr. Mater..

[16] Lee M., Kim J.-H., Park J., Kim J., Kim W., Kim D. (2004). Fabrication of Ni–Nb–Ta metallic glass reinforced Al-based alloy matrix composites by infiltration casting process. Scr. Mater..

[17] Ding H., Zhang Q., Yao K. (2024). Composition Design Strategy for High Entropy Amorphous Alloys. Materials.

[18] Lu Z., Liu C., Porter W. (2003). Role of yttrium in glass formation of Fe-based bulk metallic glasses. Appl. Phys. Lett..

[19] Su S., Ning Z., Huang Y., Yang T., Wang K., Jiang M., Sun J., Jiang S. (2022). Effect of strain rate on fracture reliability of Cu_45_Zr_45_Co_10_ amorphous alloy microwires by statistical analyses. J. Alloys Compd..

[20] Beck F., Rigue J., Carara M. (2017). The profile of the domain walls in amorphous glass-covered microwires. J. Magn. Magn. Mater..

[21] Zhukov A., Ipatov M., Del Val J., Churyukanova M., Zhukova V. (2018). Tailoring of magnetic properties of Heusler-type glass-coated microwires by annealing. J. Alloys Compd..

[22] Nematov M., Baraban I., Yudanov N., Rodionova V., Qin F., Peng H.-X., Panina L. (2020). Evolution of the magnetic anisotropy and magnetostriction in Co-based amorphous alloys microwires due to current annealing and stress-sensory applications. J. Alloys Compd..

[23] Franczak A., Levesque A., Coïsson M., Li D., Barrera G., Celegato F., Wang Q., Tiberto P., Chopart J.P. (2014). Magnetic properties dependence on the coupled effects of magnetic fields on the microstructure of as-deposited and post-annealed Co/Ni bilayer thin films. J. Magn. Magn. Mater..

[24] Gupta P., Akhila K., Srihari V., Svec P., Kane S., Rai S., Ganguli T. (2019). On the origin of magnetic anisotropy of FeCo (Nb) B alloy thin films: A thermal annealing study. J. Magn. Magn. Mater..

[25] Chen W. (1997). Asymmetric Hysteresis Loops in Amorphous Alloys. Met. Funct. Mater..

[26] Liu J., Woodcock T.G., Scheerbaum N., Gutfleisch O. (2009). Influence of annealing on magnetic field-induced structural transformation and magnetocaloric effect in Ni–Mn–In–Co ribbons. Acta Mater..

[27] Li Z., Zhang G. (1994). Displaced Hysteresis Loops of Fe-based Nanocrystalline Alloy. Met. Funct. Mater..

[28] Wang Y., Wang Q., Zhao J., Dong C. (2010). Ni–Ta binary bulk metallic glasses. Scr. Mater..

[29] Miracle D., Senkov O. (2003). Topological criterion for metallic glass formation. Mater. Sci. Eng. A.

[30] Chen S., Tu J., Wu J., Hu Q., Xie S., Zou J., Zeng X. (2016). Phase separation and significant plastic strain in a Zr–Cu–Ni–Al–Fe bulk metallic glass. Mater. Sci. Eng. A.

[31] Aich P., Meneghini C., Tortora L. (2023). Advances in Structural and Morphological Characterization of Thin Magnetic Films: A Review. Materials.

[32] Whittle G., Stewart A., Kaiser A. (1986). Magnetic and electrical properties of (FeTM) _85_B_15_ (TM= Cr, Ni) metallic glasses. Phys. Status Solidi (A).

[33] Sagasti A., Palomares V., Porro J.M., Orúe I., Sánchez-Ilárduya M.B., Lopes A.C., Gutiérrez J. (2019). Magnetic, magnetoelastic and corrosion resistant properties of (Fe–Ni)-based metallic glasses for structural health monitoring applications. Materials.

[34] Zhukova V., Ipatov M., Talaat A., Blanco J., Churyukanova M., Taskaev S., Zhukov A. (2018). Effect of stress-induced anisotropy on high frequency magnetoimpedance effect of Fe and Co-rich glass-coated microwires. J. Alloys Compd..

[35] Jacquelin J. (1993). A reliable algorithm for the exact median rank function. IEEE Trans. Electr. Insul..

[36] Elgueta M., Kittl P. (2007). Probabilistic control of materials by a simulation method. Mater. Des..

[37] Abrosimova G., Chirkova V., Pershina E., Volkov N., Sholin I., Aronin A. (2022). The Effect of Free Volume on the Crystallization of Al87Ni8Gd5 Amorphous Alloy. Metals.

[38] Jiménez-Marín E., Villalpando I., Trejo-Valdez M., Cervantes-Sodi F., Vargas-García J.R., Torres-Torres C. (2017). Coexistence of positive and negative photoconductivity in nickel oxide decorated multiwall carbon nanotubes. Mater. Sci. Eng. B.

[39] Zhang M., Qu G., Liu J., Pang M., Wang X., Liu R., Cao G., Ma G. (2021). Enhancement of Magnetic and Tensile Mechanical Performances in Fe-Based Metallic Microwires Induced by Trace Ni-Doping. Materials.

